# Interface Optimization and Performance Enhancement of Er_2_O_3_-Based MOS Devices by ALD-Derived Al_2_O_3_ Passivation Layers and Annealing Treatment

**DOI:** 10.3390/nano13111740

**Published:** 2023-05-26

**Authors:** Qiuju Wu, Qing Yu, Gang He, Wenhao Wang, Jinyu Lu, Bo Yao, Shiyan Liu, Zebo Fang

**Affiliations:** 1 Zhejiang Engineering Research Center of MEMS, Shaoxing University, Shaoxing 312000, China; wuqiuju97@outlook.com (Q.W.);; 2 Semiconductor Manufacturing Electronics (Shaoxing) Corporation, Shaoxing 312000, China; 3 School of Materials Science and Engineering, Anhui University, Hefei 230601, China; hegang@ahu.edu.cn (G.H.);

**Keywords:** Er_2_O_3_ thin film, interface state density, conduction mechanisms, high-k gate dielectric

## Abstract

In this paper, the effect of atomic layer deposition (ALD)-derived Al_2_O_3_ passivation layers and annealing temperatures on the interfacial chemistry and transport properties of sputtering-deposited Er_2_O_3_ high-k gate dielectrics on Si substrate has been investigated. X-ray photoelectron spectroscopy (XPS) analyses have showed that the ALD-derived Al_2_O_3_ passivation layer remarkably prevents the formation of the low-k hydroxides generated by moisture absorption of the gate oxide and greatly optimizes the gate dielectric properties. Electrical performance measurements of metal oxide semiconductor (MOS) capacitors with different gate stack order have revealed that the lowest leakage current density of 4.57 × 10^−9^ A/cm^2^ and the smallest interfacial density of states (Dit) of 2.38 × 10^12^ cm^−2^ eV^−1^ have been achieved in the Al_2_O_3/_Er_2_O_3_/Si MOS capacitor, which can be attributed to the optimized interface chemistry. Further electrical measurements of annealed Al_2_O_3/_Er_2_O_3_/Si gate stacks at 450 °C have demonstrated superior dielectric properties with a leakage current density of 1.38 × 10^−9^ A/cm^2^. At the same, the leakage current conduction mechanism of MOS devices under various stack structures is systematically investigated.

## 1. Introduction

Metal oxide semiconductor (MOS) capacitors are used in many different fields, such as optoelectronics, microelectronics, and biomedical diagnosis, for their excellent optical and electrical properties. However, with the increase in circuit integration, the feature size of metal oxide semiconductor field effect transistors (MOSFETs) must be reduced to less than 1 nm, which leads to a sharp deterioration in the performance of the previous MOSFETs with SiO_2_ gate dielectric due to the reduction in the size of SiO_2_ to its physical limits. The great leakage current brings about a sharp increase in the static power consumption of the logic circuit, as well as high-frequency dispersion, poor reliability, increased errors, and other problems [1]. Direct electron tunneling of SiO_2_ films with thicknesses less than 1 nm has been reported to result in severe leakage currents. Therefore, high-k gate dielectric is sought to increase the physical thickness and reduce the leakage current instead of SiO_2_ [2,3,4].

In the past decades, to decrease the leakage current and keep gate capacitance per unit area constant, some high-k gate dielectrics have been adopted to substitute SiO_2_ as the gate dielectric for MOS capacitors [5,6,7,8]. Among these candidates, rare earth oxides have gained attention due to their high dielectric constant and large band gap. Er_2_O_3_, as a classic rare earth oxide, is one of the most promising gate dielectrics to replace SiO_2_ for its large band gap (5.8 eV), large valence band offset (3.5 eV), suitable high-k value (8–20) [9], and good chemical stability when in contact with Si substrates. Moreover, Er_2_O_3_ remains amorphous when annealed at 700 °C, and its good thermal stability reduces the formation of silicide and surface roughness [10]. The gate dielectric is the most important part of MOS capacitors because the electrical characteristics of MOS capacitors depend mainly on the process parameters of the deposited film, the quality of the substrate/oxide interface, and the chemical stability of the interface. So far, the commonly used methods to obtain Er_2_O_3_ thin films include atomic layer deposition (ALD), chemical vapor deposition (CVD), and molecular beam epitaxy (MBE). The cost and environmental pollution are comparatively high for the above methods. In the current work, sputtering was carried out to sputter metallic Er targets under vacuum conditions to obtain Er_2_O_3_ films with appropriate rates at low cost and no contamination. Due to the high water vapor absorption characteristics of rare earth oxides, low dielectric constant hydroxides may be generated at the interface, resulting in reduced reliability and electrical performance of the device [11]. The water absorption of rare earth oxides may also be caused by oxygen vacancies in the film [12]. Based on previous investigations, it can be noted that aluminum oxide dielectric prepared by ALD has excellent thermal stability, oxidation resistance, and mature technology, making it an ideal passivation layer [13]. Meanwhile, the thermal annealing method is adopted to decrease the hydroxyl content in the films. Although the dielectric properties of Er_2_O_3_ gate insulator have been investigated, the effect of the location of the ALD-driven Al_2_O_3_ passivation layer and different annealing temperatures on the electric performance and interfacial bonding states of Si-based MOS capacitors have not been systematically investigated.

In this work, Er_2_O_3_ thin films were deposited using magnetron sputtering. Al_2_O_3_ passivation layers were obtained with ALD equipment, and four different gate stacks were constructed on Si, corresponding to Er_2_O_3_/Si, Er_2_O_3_/Al_2_O_3_/Si, Al_2_O_3_/Er_2_O_3_/Si, and Al_2_O_3_/Er_2_O_3_/Al_2_O_3_/Si, labeled S1, S2, S3, S4, respectively. Finally, Al_2_O_3_/Er_2_O_3_/Si gate stack was annealed from room temperature to 550 °C. Measurements were performed using X-ray photoelectron spectroscopy (XPS), capacitance-voltage (C–V), leakage current density-voltage (J–V), and conductance-voltage (G–V). The variation of the chemical composition and electrical properties of the apparent interface with the position of the alumina passivation layer and the effect of annealing temperature were systematically investigated. Three leakage current conduction mechanisms for silicon-based MOS capacitors with different passivation layer positions measured at 277 K were also investigated.

## 2. Experimental Section

**Figure 1:**
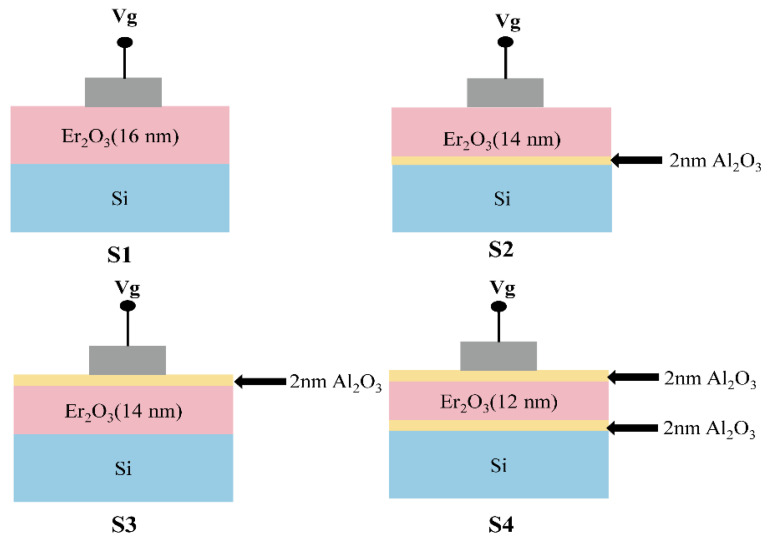
Schematic diagram of Si-based MOS capacitors based on different gate dielectric stacking structures, S1(Er_2_O_3_/Si), S2(Er_2_O_3_/Al_2_O_3_/Si), S3(Al_2_O_3_/Er_2_O_3_/Si), S4(Al_2_O_3_/Er_2_O_3_/Al_2_O_3_/Si).

N-type silicon wafers (100) with doping concentration of 1 × 10^15^ cm^−3^ and resistivity of 1–10 Ω·cm have been selected as the substrate in this study. The Si substrates were ultrasonically cleaned for 10 min at 75 °C in a mixed solution of alcohol and ammonia with H_2_O:NH_3_·H_2_O:H_2_O_2_ = 7:2:1 concentration ratio before depositing Er_2_O_3_ gate dielectric to remove organic contaminants and alkali metal impurities from the surface of the substrates. Then, wafers were rinsed with deionized water and blown dry with high-purity nitrogen. Finally, the as-processed wafers were immediately transferred from sputtering chamber to the ALD system. A total of 2 nm Al_2_O_3_ passivation layers were deposited on Si substrate by ALD, using H_2_O and trimethylaluminum (TMA) as the oxidant and Al metal precursor, respectively. After ALD Al_2_O_3_ passivation, the wafers were transferred to a sputtering chamber to deposit Er_2_O_3_ gate dielectrics by sputtering the Er target with purity of 99.9% at an operating pressure of 0.6 Pa, argon/oxygen = 50/10 SCCM, and a sputtering power of 15 W, respectively. To explore the electrical characteristics of Er_2_O_3_/Si MOS capacitor with a different stacking position of the Al_2_O_3_ passivation layer, Al electrodes with a diameter of 200 μm were deposited by thermal evaporation. Metal Al was thermally evaporated on the back side to form back ohmic contacts. Figure 1 shows the schematics of Si-based capacitors with different stacked gate dielectric structures. Sample S1 corresponds to Er_2_O_3_(16 nm)/Si, sample S2 corresponds to Er_2_O_3_(14 nm)/Al_2_O_3_(2 nm)/Si, sample S3 corresponds to Al_2_O_3_(2 nm)/Er_2_O_3_(14 nm)/Si, and sample S4 corresponds to Al_2_O_3_(2 nm)/Er_2_O_3_(12 nm)/Al_2_O_3_(2 nm)/Si, respectively. To gain high-quality films, the Al_2_O_3_/Er_2_O_3_/Si stacked device was selected for rapid thermal annealing (RTA) in a high vacuum of 5.0 × 10^−4^ Pa at 350 °C–550 °C for 2 min. The initial temperature of the rapid annealing furnace was 20 °C, and after rising to the set temperature at a rate of 10 °C/S, the temperature was maintained for 120 S, and then lowered to room temperature. XPS measurements were performed to investigate the interface chemistry of Er_2_O_3_/Si gate stacks as functions of the Al_2_O_3_ passivation layer by using the ESCALAB 250Xi system with Al Ka (1486.7 eV). The capacitance-voltage (C–V), transconductance-voltage (G–V), and leakage current-voltage (I–V) measurements were performed with the semiconductor analysis equipment (Agilent B1500A) matching with the Cascade Probe Station at room temperature. All electrical-related measurements were carried out in a dark and electrically shielded environment.

## 3. Results and Discussion

### 3.1. Interface Chemistry Analyses

**Figure 2:**
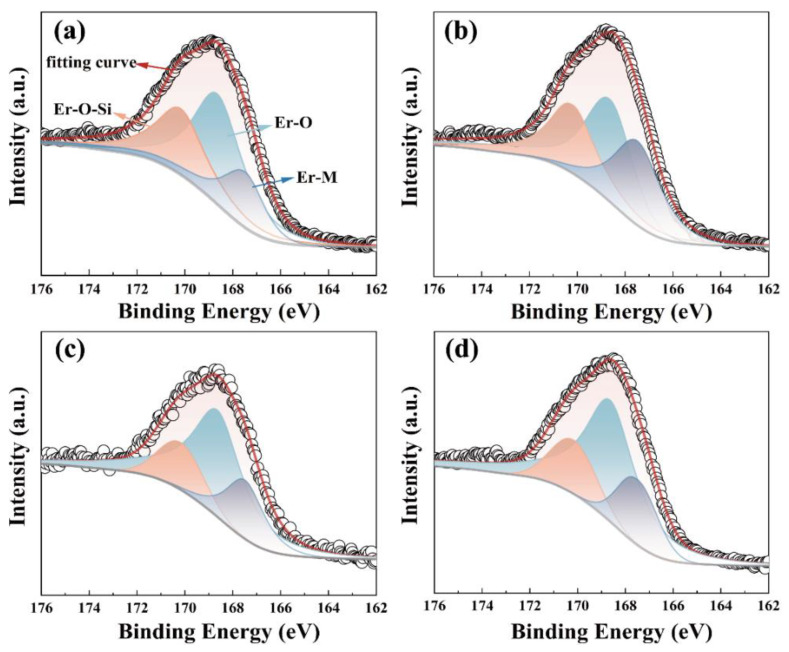
Er 4d XPS spectra of (**a**) Er_2_O_3_/Si, (**b**) Er_2_O_3_/Al_2_O_3_/Si, (**c**) Al_2_O_3_/Er_2_O_3_/Si and (**d**) Al_2_O_3_/Er_2_O_3_/Al_2_O_3_/Si.

XPS measurements have been carried out to analyze the influence of different stack orders of ALD-derived Al_2_O_3_ passivation layers on the interfacial chemical bonding states of the Er_2_O_3_/Si gate stack. Figure 2 shows the Er 4d spectra of Er_2_O_3_/Si gate stacks with different Al_2_O_3_ stacking orders. As shown in Figure S1, all elements are detected. The spectra taken from the S1, S2, S3, and S4 samples are decomposed into three different components. The peaks located at 167.49 eV can be attributed to the Er-M bonding states (M = Er, Al, Si). The peaks centered at 168.64 and 170.18 eV originate from Er-O and Er-O-Si bonds [14,15], respectively. The intensity of each component is different for each sample. It is worth noting that it is impossible to segregate the peaks of hydroxyl and silicate due to their very close binding energies, and here, ErSi(OH)_x_ is also included in Er-O-Si.

To compare more intuitively how much of each component is present, the corresponding content values are given in Table 1. S1 sample contains the highest silicate concentration, compared to the other samples, suggesting that the addition of Al_2_O_3_ can effectively prevent the diffusion of oxygen from the air via the high-k layer of the substrate. Meanwhile, it is also obvious that the silicate content of the S3 and S4 samples is almost half of those of the S1 and S2 samples. The difference among them is that S3 and S4 samples have deposited Al_2_O_3_ in the topmost layer. Due to the rare earth oxide hygroscopicity, the top Er_2_O_3_ layer will lead to the formation of the low dielectric constant hydroxides, thus causing a sharp increase in the silicate content of S1 and S2 samples. As mentioned earlier, ErSi(OH)_x_ may also be included in the silicate, which could be the reason for the difference in their silicate contents. The silicate content of S2 samples decreases by only 4% compared to S1 sample, suggesting that direct deposition of Al_2_O_3_ on the substrate may not be the best choice for the passivation of Si-based rare earth oxide interfaces.

**Figure 3:**
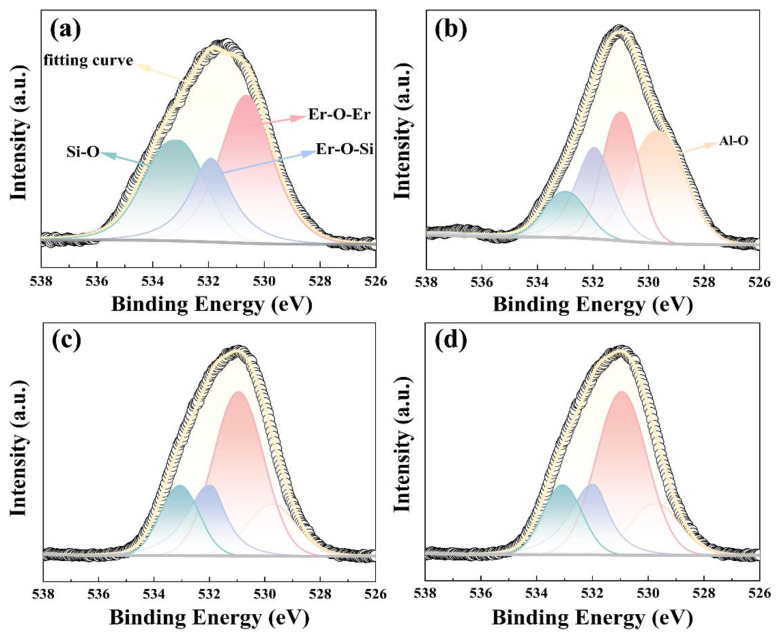
O 1S XPS spectra of (**a**) Er_2_O_3_/Si, (**b**) Er_2_O_3_/Al_2_O_3_/Si, (**c**) Al_2_O_3_/Er_2_O_3_/Si and (**d**) Al_2_O_3_/Er_2_O_3_/Al_2_O_3_/Si.

The O 1s core-level XPS spectra of all samples shown in Figure 3 are deconvoluted into four components. The first peak at 529.68 eV is caused by the Al-O bond in Al_2_O_3_. The second peak at 530.56 eV is attributed to the Er-O-Er bonds in Er_2_O_3_. It is worth noting that the Er-O-Er binding energy of S1 sample is slightly smaller than the other three samples, which may be caused by the relatively high defect density of the S1 sample, which affects the binding energy magnitude [16]. The third binding energy peak is from the Er-O-Si bond in silicate at 531.95 eV, and the highest binding energy peak is from the Si-O bond in SiO_2_, corresponding to 533.02 eV [17,18]. The positions of the individual peaks extracted from O 1s are given in Table 2. It can be found that, under the stacking conditions of S3 (Al_2_O_3_/Er_2_O_3_/Si), a decrease in the strength of the Si-O and Er-O-Si bonds, as well as an increase in the strength of the Er-O-Er bonds, has been observed. The silicate contents of S1 and S2 samples are about twice that of S3 and S4 samples. The evolution tendency of the O 1s XPS spectra is similar to the Er 4d spectra, indicating that Al_2_O_3_ passivation layer deposited on the topmost layer can better protect the device and reduce the formation of silicate to prevent the deterioration of device performance.

### 3.2. Electrical Characteristics Analysis

**Figure 4:**
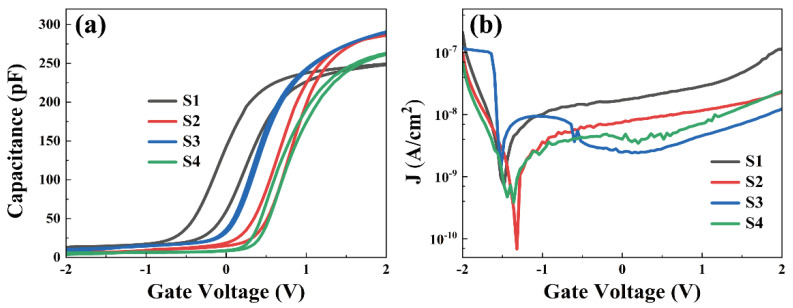
(**a**) Capacitance-voltage (C–V) curves of all samples at high frequency (1 MHz) and (**b**) leakage current density (J–V) curves of all samples.

The C–V characteristic curves of all samples measured at high frequencies (1 MHz) are illustrated in Figure 4a, demonstrating a double sweep mode from inversion to accumulation. Error plots of the J–V images of the S1–S4 samples are shown in Figure S2. Based on Figure 4, it can be seen that S1 sample has the smallest accumulation capacitance (C_ox_) and the largest hysteresis voltage, which may be caused by the low-k interface layer that occurs due to the presence of more Si oxides on the substrate. This corresponds to the maximum silicate content of S1 sample in previous XPS results. Compared with the S1 sample, optimized C–V characteristics have been observed in the sample with the passivated layer, including a significant increase of capacitance in the accumulation region and a reduction in the hysteresis and stretching phenomena, revealing that a passivation layer of Al_2_O_3_ passivated layer significantly suppresses the generation of low-k interfacial layers, thus weakening the interfacial Fermi pinning effect. In particular, compared with samples S2 and S4, the capacitance of the accumulation region of sample S3 is more saturated and the hysteresis is smaller, and sample S3 has the best electrical properties, which suggests that the passivation treatment has effectively controlled the interfacial trap density and slow interface states density [19]. As shown in Table 3, some important electrical parameters extracted from the C–V curves can help us in the quantitative analysis of the electrical properties of the silicon-based MOS capacitors. Also, in Figures S3 and S4, a line graph is used to more visually represent the data. The k values of the four samples are calculated to be 12.49, 14.61, 14.70, and 13.25. It is observed that S3 has the largest dielectric constant. Table 3 also displays the values of flat band voltage (*V_fb_*), hysteresis voltage (Δ*V_fb_*), the density of oxide charges (*Q_ox_*), and the border-trapped oxide charge (*N_bt_*) of all deposited samples. The flat-band voltage of the MOS capacitor is 0 V in the ideal state, but the flat-band voltages of our measured samples are −0.33, 0.41, 0.15, and 0.47 V, respectively, which can be due to the presence of the oxide charges located in the oxide layer or at the interface between the oxide layer and the semiconductor. Before the induction of the passivation layer, the *V_fb_* of the sample is negative, indicating that a positive oxide charge is created in Er_2_O_3_ film. After the introduction of the Al_2_O_3_ passivation layer, the positive *V_fb_* indicates that the passivation layer probably brings some oxygen vacancies, which can trap electrons to form negatively charged centers [20]. The oxide charge mainly includes fixed oxide charge (*Q_f_*), movable charge (*Q_m_*), oxide trap charge (*Q_ot_*), and interface trap charge (*Q_it_*). *Q_f_* is generated mainly because of oxygen vacancies formed during the oxidation process. The Al_2_O_3_/Er_2_O_3_/Si stacked structure of S3 sample has the smallest *V_fb_* value (0.15 V), which proves that it can effectively reduce the oxidation charge defects. As the oxygen vacancy at the interface diminishes, the flat-band voltage also decreases [21]. This is also evidenced by the smallest oxide charge density *Q_ox_* (−7.21 × 10^11^ cm^−2^) of the S3 sample, which probably occurs because the Al_2_O_3_ passivation layer deposited on the topmost part effectively isolates the air from contact with the gate oxide and the substrate, and the extremely low oxygen diffusion of the Al_2_O_3_ layer avoids the introduction of new impurity defects and natural oxides.

The difference between the *V_fb_* of the C–V curve in two directions with double-sweep mode is the Δ*V_fb_*. The magnitude of Δ*V_fb_* is associated with the boundary-captured oxide charge (*Q_bt_*) [22]. Compared to the control S1, the hysteresis voltage of the sample with the added passivation layer is significantly reduced, indicating a significant decrease in the boundary trapping charge density, while the almost zero hysteresis voltage of the S3 sample proves that the top wrapped structure is more effective in reducing defects and protecting the device. Based on the measured values of *V_fb_* and Δ*V_fb_*, the associated *Q_ox_* and *N_bt_* values are determined by the following equations [23].

(1) $$Q_{ox}=-C_{max}\left(V_{fb}-\Phi_{ms}\right)/qA$$

(2) $$N_{bt}=-\left(C_{max}\times \Delta V_{fb}\right)/qA$$

where *Φ_ms_* is the contact potential difference between the Al electrode and Si substrate, *q* is the fundamental charge, and A is the area of the Al electrode. The lowest *Q_ox_* value (−7.21 × 10^11^ cm^−2^) and the lowest *N_bt_* value (−1.80 × 10^11^ cm^−2^) visualize the optimized electrical performance of the Al_2_O_3_/Er_2_O_3_/Si gate stacking junction of the S3 sample.

**Figure 5:**
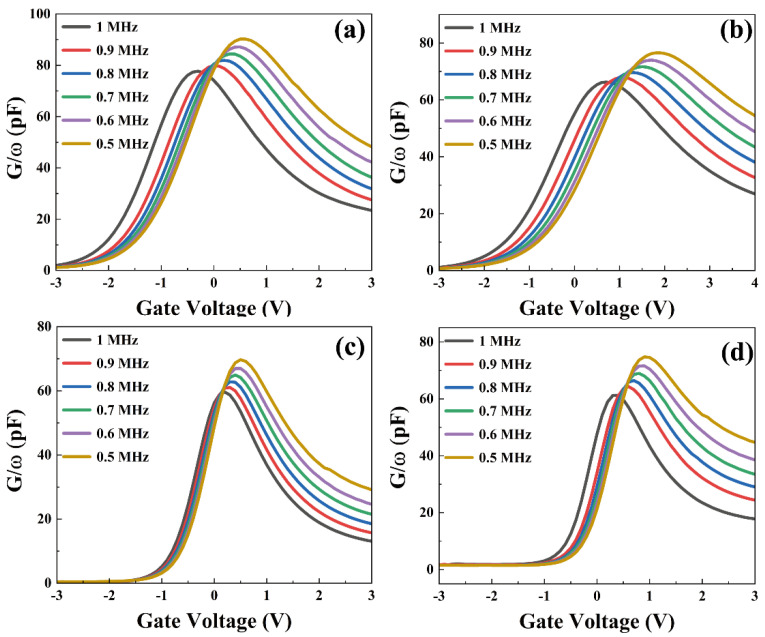
Multi-frequency G/ω-V characteristic curves of (**a**) S1, (**b**) S2, (**c**) S3, and (**d**) S4 samples.

To characterize the interfacial state density quantitatively and show the distribution in the trap energy level, Figure 5a–d show the conductance/angular frequency-voltage (G/ω-V) curves of the samples measured at 500 kHz–1 MHz. The relationship between the interface trap capacitance and the interface density of states is *C_it_* = *qD_it_*. Interfacial traps around the Fermi energy level could shift their occupancy and change the conductivity, producing regular changes. The apparent shift in the location of the peak conductivity indicates the effectiveness of the Fermi energy level shift. The conductivity approach is based on the analysis concerning losses due to changes in the charge state of trap energy levels. The largest loss takes place when the interface trap and the applied AC signal move in resonance (*ωτ* = 1), the frequency dependence is associated with the response time of the characteristic trap, *τ* = *2π/ω*, and the capture and emission rates from the Shockley-Read-Hall theory modulate the response time [24].

(3) $$\tau =\frac{exp\left(\Delta E/k_{B}T\right)}{\sigma \upsilon_{th}D_{dos}}$$

where Δ*E* is the energy difference between the trap energy level *E_T_* and the edge of the majority carrier energy band, *k_B_* is the Boltzmann constant, *T* is the temperature, *υ_th_* is the average speed of the majority carrier produced by thermal excitation, *D_dos_* is the effective density of states of the majority carrier energy band, and *σ* is the trapping cross-section of the trap [25]. The significant shift of the peak in Figure 5 indicates that the fabricated devices have few interfacial defects and a low degree of interfacial Fermi energy level pinning. Under the assumption that the surface potential could be neglected, *D_it_* can be evaluated from the normalized parallel conductance peak (*G_P_*/*ω*)*_max_* [26].

(4) $$D_{it}\approx \frac{2.5}{Aq}{\left(\frac{G_{P}}{\omega }\right)}_{max}$$

Here, *A* is the area of the device. To determine the position of the trap energy level *E_T_*, the difference between the *E_T_* and the *E_c_*, that is, the energy band bending potential of *E_T_*, needs to be determined. Where the value of *E_T_* could be obtained from the frequencies in (*G_P_*/*ω*)*_max_*. Using the above formula to calculate *D_it_*, with correspondence to Δ*E* [27].

(5) $$\Delta E=\left(E_{c}-E_{T}\right)=\frac{k_{B}T}{q}ln\left(\frac{\sigma \upsilon D_{dos}}{2\pi f_{max}}\right)$$

**Figure 6:**
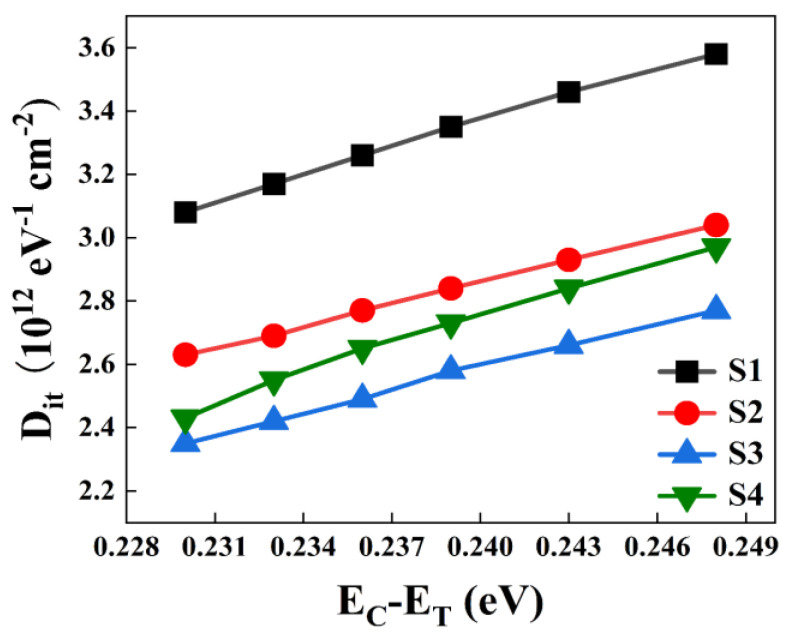
Relationship between the energy levels of samples S1–S4 and the extracted *D_it_*.

According to the conductivity method, the *D_it_* data of each sample was obtained, as displayed in Figure 6. It is clear that, as *E_c_*−*E_T_* increases, the *D_it_* of each sample also increases gradually. Meanwhile, the overall defect density of S3 sample is the lowest, compared with the other three samples. Experimental results have shown that the ALD-derived Al_2_O_3_ passivation layer on the topmost layer of the device can effectively inhibit the formation of Er(OH)_x_ or silicate and reduce the interfacial density of states, thus preparing high-quality Si-MOS capacitors. Compared with S1, the average *D_it_* value of S3 samples was reduced from 3.08 × 10^12^ eV^−1^ cm^−2^ to 2.35 × 10^12^ eV^−1^ cm^−2^.

The conduction mechanism in dielectric films is the key to the successful application of dielectric materials. Different leakage current conduction mechanisms can cause significant differences in leakage currents. To explore the effects of different gate dielectric stacking methods on carrier transport mechanisms, three current conduction mechanisms (CCMs) are systematically discussed under substrate injection including Schottky Emission (SE), Poole–Frenkel Emission (PF) and Fowler–Nordheim Tunneling (FN) [28].

SE emission is a very classical form of thermal ionization emission where a charge gains energy under the action of an applied electric field and overcomes the potential barrier between the metal electrode and the gate dielectric, forming a leakage current. SE emission is usually found at high temperatures, and *SE* is formulated as follows.

(6) $$J_{SE}=A^{*}T^{2}exp\left[\frac{-q(\varphi_{B}-\sqrt{qE/4\pi \varepsilon_{0}\varepsilon_{r}}}{k_{B}T}\right]$$

(7) $$A^{*}=\frac{4\pi qk_{B}^{2}m_{ox}^{*}}{h^{3}}=120\frac{m_{ox}^{*}}{m_{0}}$$

**Figure 7:**
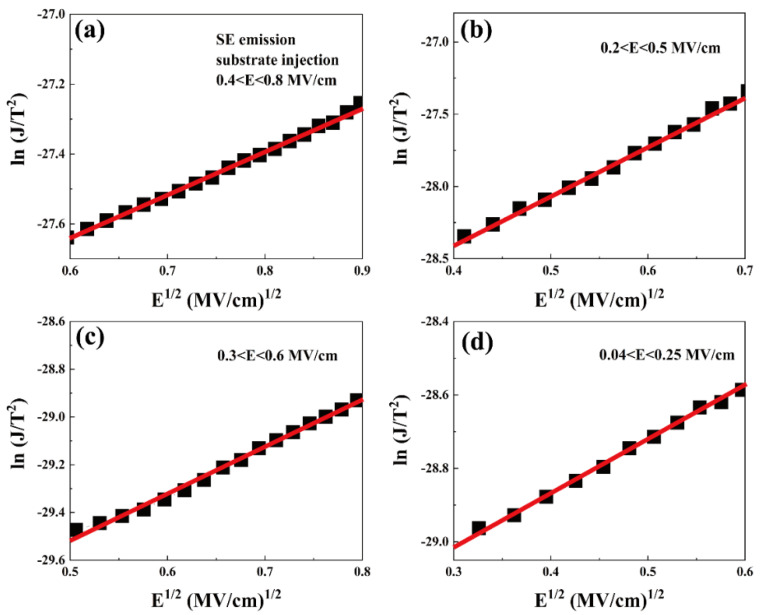
SE emission fitting curves of substrate-injected samples (**a**) S1, (**b**) S2, (**c**) S3, and (**d**) S4 at 277 K.

Among them, A* represents the effective Richardson constant, *m_o_* is the mass of free electrons, *m_ox_* is the effective mass of electrons in the gate oxygen layer, *φ_B_* is the Schottky barrier height, and E is the applied electric field, which could be determined by using the equation: *E* = (*V* − *V_fb_*)/*t_ox_*. *ε*_0_ and *ε_r_* denote the vacuum dielectric constant and the optical dielectric constant, respectively, and k_B_ is the Boltzmann constant [29]. The optical permittivity *ε_r_* is meant to be close to the square of refractive index (*n* = *ε_r_*^1/2^ = 1.95). For a standard SE emission, *E*^1/2^ and ln(J/T^2^) are in a proportional relationship. Figure 7 presents good linear fits of ln(J/T^2^) and E^1/2^ at a low field region (0.1–0.8 MV/cm), the slope of the SE-fitted curve is:

(8) $$\begin{array}{c} slope=\sqrt{\frac{q^{3}}{4\pi \varepsilon_{0}\varepsilon_{r}}}/k_{B} \end{array}$$

Based on the relationship between n and *ε_r_*, the calculated n values of S1, S2, S3, and S4 are 12.0, 4.4, 7.4, and 10.0, respectively, which are very different from the expected value of 1.95, indicating that there are other mechanisms dominating the current flow through the films.

**Figure 8:**
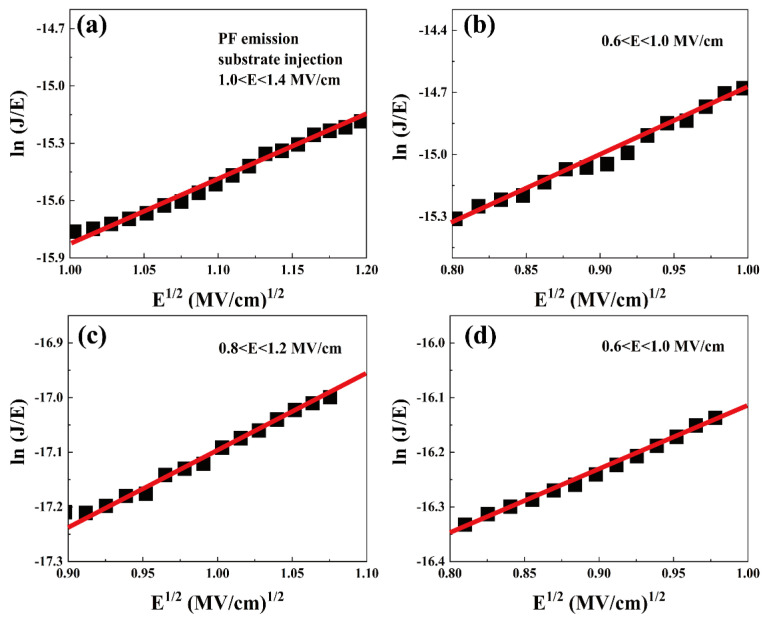
PF emission fitting curves of substrate-injected samples (**a**) S1, (**b**) S2, (**c**) S3, and (**d**) S4 at 277 K.

PF emission mechanism is attributed to the phenomenon of thermally excited electrons gaining enough energy to jump between the traps in the dielectric layer, forming a gate leakage current in the conduction band of the gate dielectric, which can be expressed by the following equation [30].

(9) $$J_{PF}=BE\;exp\left[\frac{-q\left(\varphi_{t}-\sqrt{qE/\pi \varepsilon_{0}\varepsilon_{ox}}\right)}{k_{B}T}\right]$$

where *φ_t_* is the trap energy level, *ε*_0_ denotes the oxide dielectric constant, and B represents a constant [31]. For standard PF emission, *E*^1/2^ and ln(*J*/*E*) should be in a good proportional relationship, as shown in Figure 8. It can be seen that PF emission mechanism at 277 K is consistent in the higher electric field region (0.6–1.4 MV/cm), but the extracted *ε_ox_* for S1, S2, S3, S4 is calculated to be 82.1, 82.6, 458, 648, respectively. The large difference from the measured value means that PF emission mechanism is not dominant at lower temperatures.

**Figure 9:**
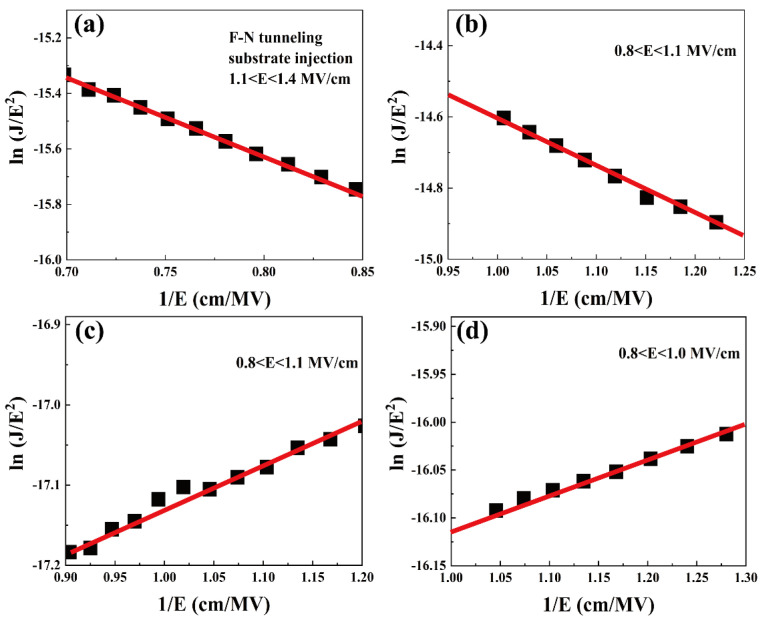
F-N tunneling mechanism of substrate-injected samples (**a**) S1, (**b**) S2, (**c**) S3 and (**d**) S4 at 277 K.

FN tunneling is a slightly temperature-dependent conduction mechanism that is usually observed at lower temperatures, that is, the process by which electrons pass directly from the gate medium and enter the conduction band, and the *J_FN_* is expressed by the following Equation [32].

(10) $$J_{FN}=\frac{q^{3}E^{2}}{16\pi^{2}\hbar \varphi_{B}}\;exp\left[-\frac{4\sqrt{2m_{T}^{*}\varphi_{B}^{3}}}{3\hbar qE}\right]$$

where $m_{T}^{*}$ is the tunneling effective mass in the gate oxide film, *φ_B_* is the potential barrier height, and the other parameters are defined as before [33]. For the FN tunneling conduction mechanism, ln(*J*/*E*^2^) and 1/*E* for all samples conforms to a linear relationship, as shown in Figure 9. The measured J–V curves for all the samples at 0.8–1.4 cm/MV are also in good agreement with FN tunneling, indicating that the lower temperature (277 K) suppresses the hot electron SE and P-F emission, while the temperature-independent FN tunneling currents can be conducted at lower temperatures; therefore, smaller currents are formed at low temperatures.

### 3.3. Annealing Dependent Electrical Characteristics Analysis

**Figure 10:**
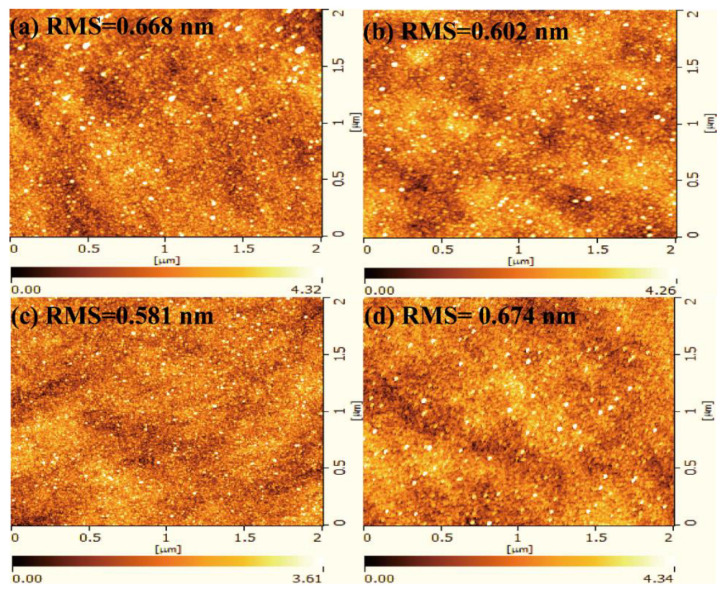
AFM of S3 samples of (**a**) as-deposited, (**b**) 350 °C-annealed, (**c**) 450 °C-annealed, (**d**) 550 °C-annealed. Scale bar = 0.5 μm.

Based on previous analyses, it can be concluded that the S3 sample (Al_2_O_3_/Er_2_O_3_/n-Si MOS capacitors) generates fewer interfacial defects and the smallest interfacial state density, indicating that the best device performance has been obtained for the Al_2_O_3_/Er_2_O_3_/Si with the appropriate passivation layer position. However, the sputtering-derived gate dielectric films do not reach the ideal state and still have a high density of interfacial states and a high leakage current density. Therefore, annealing of the sample should be carried out to reduce the number of defects and impurities in the film. Nevertheless, with increasing annealing temperature, the morphology of the erbium oxide film may change, and silicates may be formed at the interface, which might lead to compromised electrical properties of MOS capacitors [34]. Therefore, it is very important to determine the optimal annealing conditions for the fabrication of high-quality devices. Figure 10 shows the AFM images of the S3 sample annealed at different temperatures from 350 °C to 550 °C. The root mean square (RMS) of the S3 sample has been calculated to be 0.668, 0.602, 0.581, and 0.674 nm, respectively. It can be seen that a suitable annealing temperature can reduce the surface roughness, and the S3 sample annealed at 450 °C has the smallest RMS value. This is mainly because a suitable annealing temperature can enhance the stoichiometric ratio of the insulator and deal with defects. Low surface roughness is conducive to the preparation of high-performance devices, and a flatter surface can effectively avoid interfacial charge generation [35]. The sample is still amorphous after annealing at 550 °C, as shown in Figure S5.

**Figure 11:**
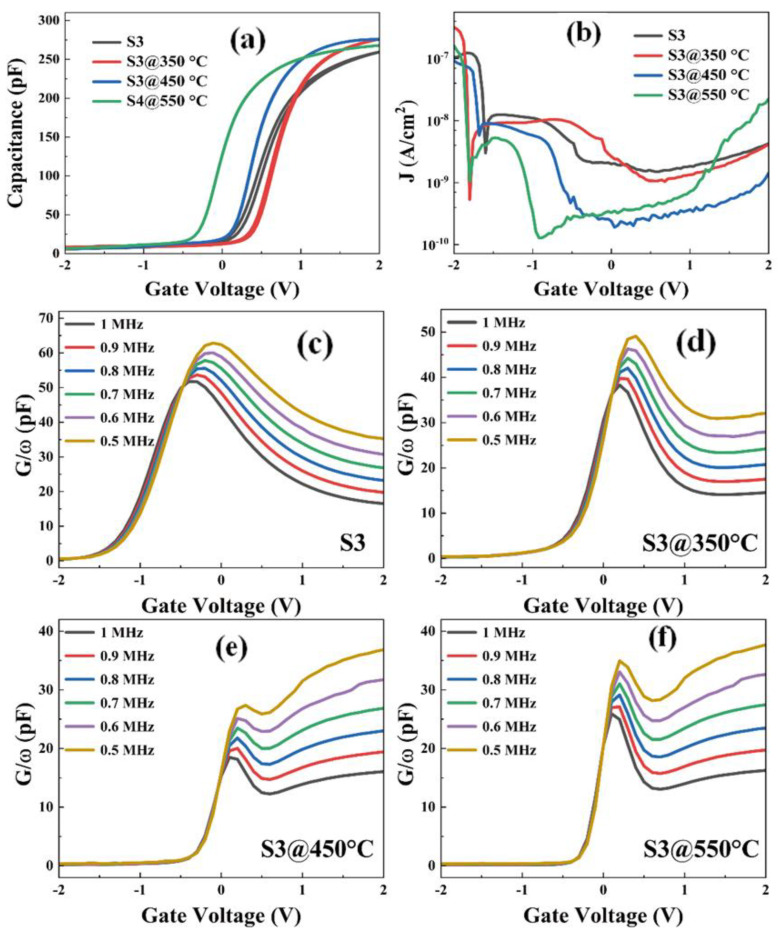
(**a**) Capacitance-voltage (C–V) and (**b**) Leakage current density-voltage (J–V) characteristic curves of Al/Al_2_O_3_/Er_2_O_3_/Si capacitors at different annealing temperatures. (**c**–**f**) Multi-frequency G/ω-V characteristic curves of Al/Al_2_O_3_/Er_2_O_3_/n-Si gate stacks annealed at different temperatures.

Figure 11a shows the C–V curves of the S3 sample annealed at different temperatures. The important electrical parameters extracted from Figure 11a are listed in Table 4. Meanwhile, in Figures S6 and S7, a line graph is used to more visually represent the data. It is evident that the *k* values increase significantly increase with the increased annealing temperature, and that all the k values are within the reported values. The capacitance of the accumulation region of the annealed samples increases from the as-deposited state to the annealed state at 450 °C, while the capacitance value decreases after annealing at 450 °C. The reduction of the capacitance may result from the thickening of the interface layer between Si and Er_2_O_3_, and higher annealing temperature may lead to the breakage of the sub-stable Er-Si bond, while ErSiO_y_ may be the major component of the interfacial layer [36]. In addition to the interfacial layer, another reason for the decrease in capacitance may be the leakage current, as the higher the leakage current, the smaller the capacitance [37]. Comparing all the parameters, it can be seen that the S3@450 °C sample obtains a smaller oxide charge density (−1.20 × 10^12^ cm^−2^), a smaller boundary trap density (−2.74 × 10^10^ cm^−2^), the largest dielectric constant (15.8), and the smallest hysteresis voltage (0.005 V) and leakage current density(3.68 × 10^−10^ A/cm^2^). This result indicates that treatment at an appropriate annealing temperature of 450 °C can further optimize the interfacial and electrical properties of S3 samples.

Figure 11b displays the J–V curves of the capacitor with the leakage current density values of 1.83 × 10^−9^, 1.33 × 10^−9^, 3.68 × 10^−10^, and 7.19 × 10^−10^A/cm^2^ for S3 and 350 °C–550 °C treated samples at 1 V, respectively. The best current-voltage characteristics are achieved for the 450 °C-annealed sample compared to the as-deposited one, which can be due to the fact that the proper annealing treatment reduces the generation of defective states at the gate dielectrics as well as at the interface, thus reducing the probability of trap-assisted current generation. Figure 11c–f show the *G/ω-V* curves of all the samples, and it can be observed that the conductivity peaks of all samples are significantly shifted, demonstrating the effectiveness of the fermi energy level shift, and that the obtained *D_it_* has a good reference value. To quantify the defective interfacial defect distribution, the interfacial density of states (*D_it_*) from *G/ω-V* curves for all samples in the frequency range from 500 kHz to 1 MHz have been extracted.

**Figure 12:**
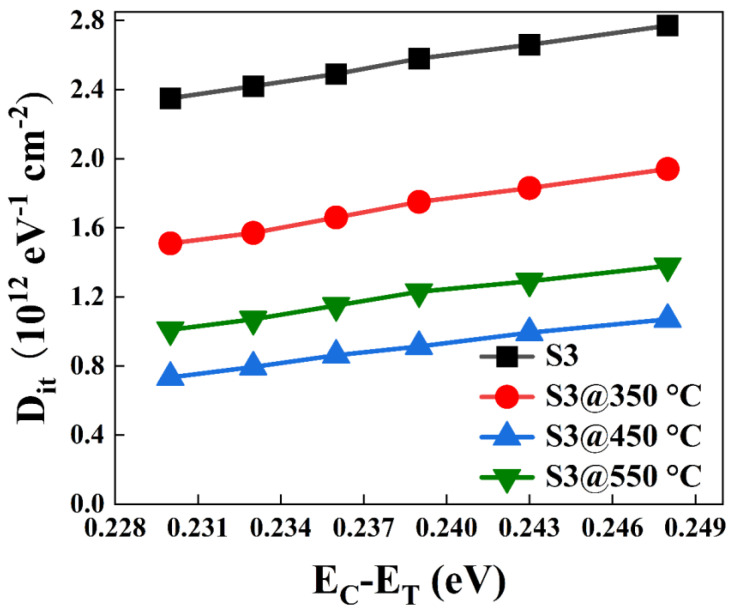
Distribution of *D_it_* for all samples at different annealing temperatures.

According to the electrical conductivity method, as shown in Figure 12, the relationship between *D_it_* distribution and ΔE has been obtained for all samples. It is observed that, with the gradual increase of E_C_−E_T_ value, the *D_it_* of each sample gradually increases, and the *D_it_* of the unannealed S3 sample is significantly larger than the other samples. The overall defect density of the S3@450 °C sample is significantly smaller than that of the other samples, indicating an optimized interfacial quality. Thus, it could be summarized that the degradation of the interfacial chemistry results in the highest *D_it_* for S3 samples, and the qualitative annealing efficacy leads to the discrepancy between S3 and S3@350 °C, S3@450 °C, and S3@550 °C samples. This is mainly because a suitable annealing temperature can effectively reduce the interfacial intrinsic oxide and silicate content, which reduces the interfacial defects. Based on the above analysis, the device at S3@450 °C achieves the best dielectric properties by reducing the interfacial state.

## 4. Conclusions

In summary, the effect of ALD-derived layered passivation layer position and annealing temperature on the interfacial chemistry and current transport characteristics of sputter-deposited Er_2_O_3_/Si gate-stacks are explored in detail. It can be found that the Al_2_O_3_/Er_2_O_3_/Si gate stack structure can significantly protect the substrate oxide from diffusion and significantly improve the MOS device’s electrical performance, including larger accumulation region capacitance, and lowest leakage current density of 4.57 × 10^−9^ A/cm^2^. Different laminated gate structures were evaluated using the density of interfacial states extracted by the conductivity method, and the results have showed that the Al_2_O_3_/Er_2_O_3_/Si gate stacks have achieved the lowest density of interfacial states at 2.35 × 10^12^ eV^−1^cm^−2^. According to the analysis of CCMs, the lower temperature (277 K) suppresses the hot electron SE and PF emission, while the temperature-independent FN tunneling current can be conducted at lower temperatures, and FN tunneling is the only predominant mechanism at the lower temperature, thus forming a smaller current at low temperatures. The annealing treatment of Al_2_O_3_/Er_2_O_3_/Si MOS capacitors at 450 °C has showed improved electrical properties, including suppression of interfacial growth, leakage current density of 1.38 × 10^−9^ A/cm^2^ at 1 V, as well as maximum dielectric constant of 15.8, and the minimum interfacial density of states of 7.4 × 10^11^ eV^−1^cm^−2^. In conclusion, both the surface passivation and heat treatment can effectively suppress the regrowth of interface species and guarantee the potential application of Al_2_O_3_/Er_2_O_3_/Si gate stacks in future microelectronics.

## Figures and Tables

**Table 1:** The contents of the peaks extracted from Er 4d XPS spectra.

	Er-M	Er-O	Er-O-Si
S1	22.10%	46.77%	31.13%
S2	30.26%	39.27%	29.83%
S3	20.85%	61.38%	17.76%
S4	19.24%	62.64%	18.12%

**Table 2:** The contents of the peaks extracted from O 1s XPS spectra.

	Al-O	Er-O-Er	Er-O-Si	Si-O
S1	-	41.06%	27.44%	31.51%
S2	36.61%	28.34%	23.32%	11.74%
S3	28.94%	44.83%	15.96%	10.37%
S4	15.54%	49.30%	18.91%	16.25%

**Table 3:** MOS capacitor’s electrical parameters obtained from C–V and J–V curves.

Sample	k	V_fb_ (V)	ΔV_fb_ (V)	Q_ox_ (cm^−2^)	N_bt_ (cm^−2^)	J (A/cm^2^)
S1	12.49	−0.33	0.34	1.80 × 10^12^	−1.72 × 10^12^	2.87 × 10^−8^
S2	14.61	0.41	0.17	−2.30 × 10^12^	−9.59 × 10^11^	1.16 × 10^−8^
S3	14.70	0.15	0.03	−7.21 × 10^11^	−1.80 × 10^11^	4.57 × 10^−9^
S4	13.25	0.47	0.10	−2.29 × 10^12^	−5.21 × 10^11^	7.14 × 10^−9^

**Table 4:** Electrical parameters of MOS capacitors at different annealing temperatures extracted from the C–V and J–V curves.

Sample	C_ox_(pF)	k	ΔV_fb_ (V)	Q_ox_ (cm^−2^)	N_bt_ (cm^−2^)	J (A/cm^2^)
S3	259	14.9	0.050	–1.75 × 10^12^	–2.73 × 10^11^	1.83 × 10^−9^
S3@350 °C	275	15.8	0.030	–2.73 × 10^12^	–1.86 × 10^11^	1.33 × 10^−9^
S3@450 °C	275	15.8	0.005	–1.20 × 10^12^	–2.74 × 10^10^	3.68 × 10^−10^
S3@550 °C	268	15.4	0.010	1.05 × 10^12^	–1.60 × 10^10^	7.19 × 10^−10^

## Supplementary Materials

The following supporting information can be downloaded at: https://www.mdpi.com/article/10.3390/nano13111740/s1. Figure S1. XPS full spectrum of S4 sample. Figure S2. XRD patterns of S3 and S3@550 °C. Figure S3. Comparison of k, V_fb_, and ΔV_fb_ values for S1–S4 samples. Figure S4. Comparison of absolute values of Q_ox_ and N_bt_ and comparison of leakage current density values for S1–S4 samples. Figure S5. Comparison of C_ox_, k, and ΔV_fb_ values for S3, S3@350 °C, S3@450 °C and S3@550 °C samples. Figure S6. Comparison of absolute values of Q_ox_ and N_bt_ and comparison of leakage current density values for S3, S3@350 °C, S3@450 °C and S3@550 °C samples. Figure S7. (a–d) are the error plots of J–V curves for S1–S4 samples.
