# Supplementary material for: Interface Optimization and Performance Enhancement of Er2O3-Based MOS Devices by ALD-Derived Al2O3 Passivation Layers and Annealing Treatment
Source: Nanomaterials (Basel). 2023 May 26;13(11):1740. doi: 10.3390/nano13111740 (PMC10254668; doi:10.3390/nano13111740)
Supplement: Supplementary file 1 [file nanomaterials-13-01740-s001.zip › nanomaterials-2391759-SI.pdf]

Supplementay Information

Interface optimization and performance enhancement of Er_2O_3-based MOS devices by ALD-derived Al_2O_3 passivation layers and annealing treatment

Q. J. Wu [1], Q. Yu [2], G. He [3,], W. H. Wang [3], J. Y. Lu [3], B. Yao [1,*], S. Y. Liu [1], Z. B. Fang [1,*]

[1] *Zhejiang Engineering Research Center of MEMS, Shaoxing University, Shaoxing 312000, PR China*

[2] *Semiconductor Manufacturing Electronics (Shaoxing) Corporation, Shaoxing 312000, PR China*

[3] *School of Materials Science and Engineering, Anhui University, Hefei 230601, PR China*

* *Correspondence: yaob_usx@163.com (B. Yao); csfzb@usx.edu.cn (Z. B. Fang)*

[*]Corresponding authors.

E-mail address: yaob@usx.edu.cn(B. Yao).

csfzb@usx.edu.cn(Z. B. Fang)

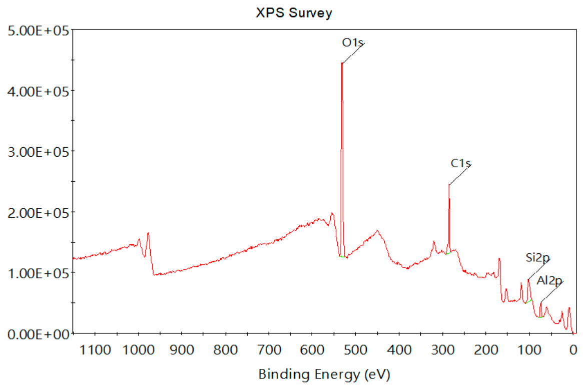

Fig. S1. XPS full spectrum of S4 sample.

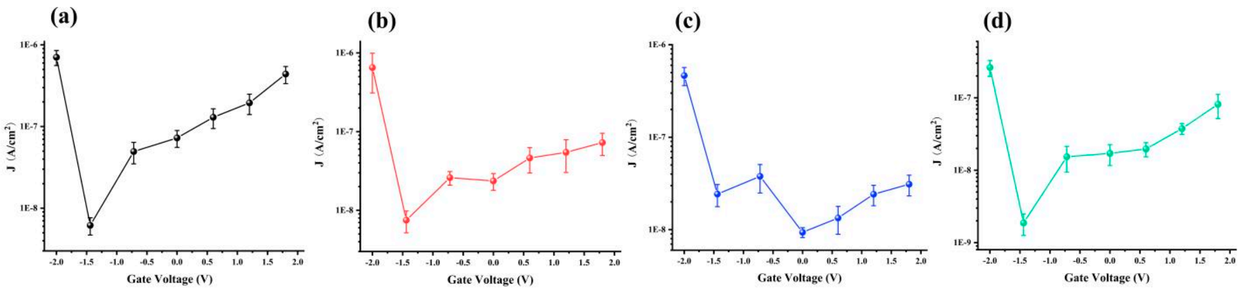

Fig. S2. (a)-(d) are the error plots of J-V curves for S1-S4 samples

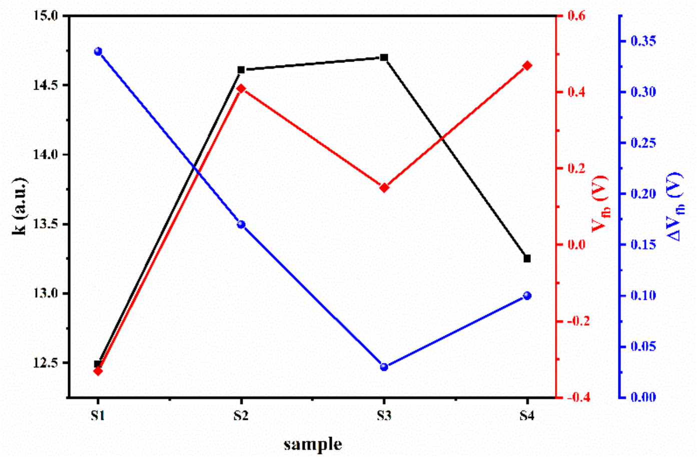

Fig. S3. Comparison of k, V_{fb} and ΔV_{fb} values for S1-S4 samples.

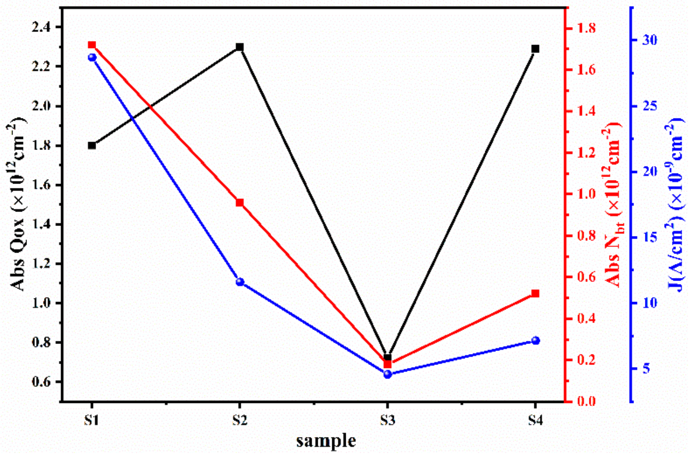

Fig. S4. Comparison of absolute values of Q_{ox} and N_{bt} and comparison of leakage current density values for S1-S4 samples.

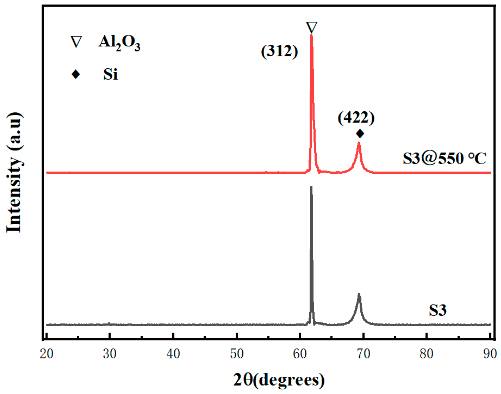

Fig. S5 XRD patterns of S3 and S3@550 ℃.

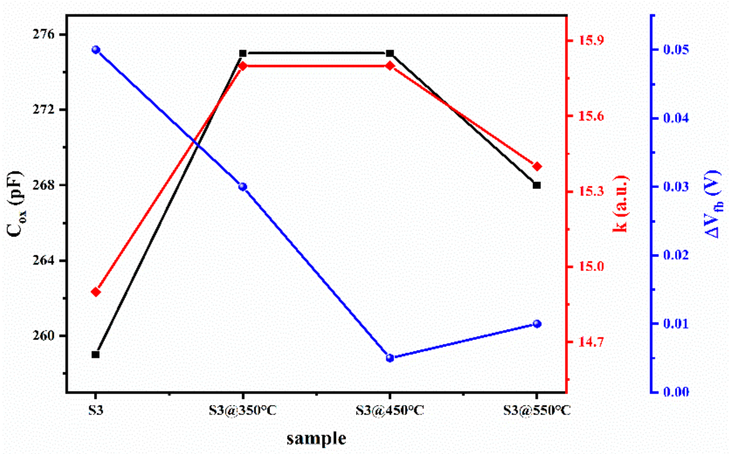

Fig. S6. Comparison of C_{ox} , k, and ΔV_{fb} values for S3, S3@350℃, S3@450℃ and S3@550℃ samples.

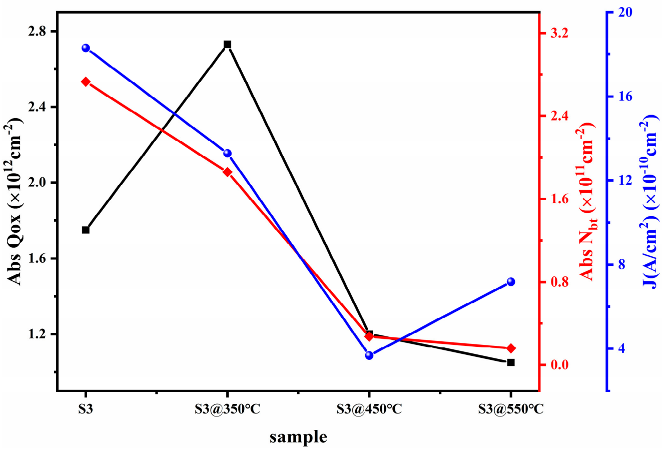

Fig. S7. Comparison of absolute values of Q_{ox} and N_{bt} and comparison of leakage current density values for S3, S3@350℃, S3@450℃ and S3@550℃ samples.
